# An Update on Antitumor Activity of Naturally Occurring Chalcones

**DOI:** 10.1155/2013/815621

**Published:** 2013-04-17

**Authors:** En-Hui Zhang, Ru-Feng Wang, Shu-Zhen Guo, Bin Liu

**Affiliations:** ^1^School of Chinese Medicine, Beijing University of Chinese Medicine, Beijing 100102, China; ^2^School of Preclinical Medicine, Beijing University of Chinese Medicine, Beijing 100029, China

## Abstract

Chalcones, which have characteristic 1,3-diaryl-2-propen-1-one skeleton, are mainly produced in roots, rhizomes, heartwood, leaves, and seeds of genera *Angelica, Sophora, Glycyrrhiza, Humulus, Scutellaria, Parartocarpus, Ficus, Dorstenia, Morus, Artocarpus*, and so forth. They have become of interest in the research and development of natural antitumor agents over the past decades due to their broad range of mechanisms including anti-initiation, induction of apoptosis, antiproliferation, antimetastasis, antiangiogenesis, and so forth. This review summarizes the studies on the antitumor activity of naturally occurring chalcones and their underlying mechanisms in detail during the past decades.

## 1. Introduction

Chalcones are one of the subclasses of flavonoid family. They are commonly known as yellow pigments in flowers, and are also widely distributed in various parts (roots, rhizomes, heartwood, buds, leaves, flowers, and seeds) of species of genera *Angelica*, *Sophora*, *Glycyrrhiza*, *Humulus*, *Scutellaria*, *Parartocarpus*, *Ficus*, *Dorstenia*, *Morus*, *Artocarpus*, and so forth. A wide range of biological activities have been attributed to chalcones, including antitumor, antimutagenic, anti-inflammatory, antioxidant, antifungal, antibacterial, antiprotozoal, analgesic, and gastric protective properties [1]. As for antitumor activity, chalcones exhibit various effects including anti-initiation, apoptosis induction, antiproliferation, antimetastasis, antiangiogenesis, and so forth. This review summarizes the studies on the antitumor activity of naturally occurring chalcones and their underlying mechanisms in detail during the past decade and also briefly categorized these compounds on the basis of their structures.

## 2. Structures

Chalcones, a term introduced by Kostanecki and Tabor [2], are considered as the precursors of flavones in the biosynthesis of flavonoids. Structurally, they are a group of compounds bearing 1,3-diaryl-2-propen-1-one framework, which consists of open-chain flavonoids in which the two aromatic rings are joined together by three carbons in an *α*,*β*-unsaturated system. Chemically, they can be easily cyclized by a Michael addition at the *β* position of the carbonyl to form a flavanone. The existence of double bond results in *cis* and *trans* isomeric forms of chalcone in nature, of which the *trans* form is thermodynamically stable. Chalcone is one of the most structurally diverse groups of flavonoids existing as dimmers, oligomers, Diels-Alder adducts, and conjugates of various kinds. Additionally, the attachment of varieties of hydroxyl, methoxy, and alkenyl functionalities to the framework of chalcone contributes to its structural diversity as well [3].

**Figure 1:**
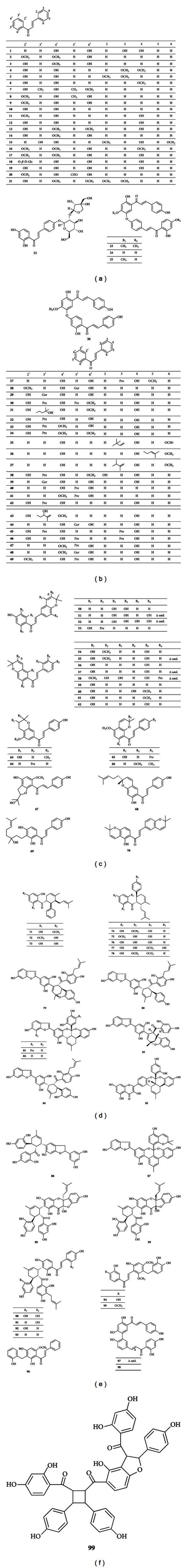
Structures of chalcones.

During the last decade, about 90 compounds of chalcones with various antitumor activities were reported. They can be roughly categorized into 4 large groups, namely, chalcones with *O*-substitution, prenylated chalcones, Diels-Alder adducts, and others. All these 90 compounds are shown in Figure 1.

### 2.1. Chalcones with *O*-Substitution

This group of compounds (**1**–**26**) carries a varying number of *O*-substituents on A-ring and B-ring, including hydroxyl and methoxy groups. These groups are usually located at C-2′, C-4′, C-6′, and C-4 and seldom appear at C-3′, C-5′, C-2, C-3, and C-6. In addition, 2 *O*-glycosylated chalcones (**18**,** 22**) as well as 4 *O*-chalcone dimmers (**23**–**26**) are also included in this group.

### 2.2. Prenylated Chalcones

Prenylated chalcones (**27**–**70**) account for a significant proportion of chalcones. In structure, various isoprenoid-derived groups can be considered as substituents to the chalcone skeleton, such as prenyl, geranyl, furano, and pyranochalcones.

### 2.3. Diels-Alder Adducts

Chalcones (**71**–**93**) fall into the category of Diels-Alder adducts. Diels-Alder reaction is an organic chemical reaction (specifically, a cycloaddition) between a conjugated diene and a substituted alkene, commonly termed dienophile, to form a substituted cyclohexene system. Chalcones act as the dienophiles in Diels-Alder reactions, and the dienes that participate in the formation of the Diels-Alder adducts range from simple isoprene and monoterpene compounds to coumarins and other classes of flavonoids [3]. 

### 2.4. Others

In addition, antineoplastic chalcones above 3 groups were also reported recently, which include 3 *C*-benzylated chalcones (**94**–**96**), 2 dimmers (**97**,**98**), and a trimer (**99**).

## 3. Mechanisms of Antitumor Activity

All chalcones illustrated in Figure 1 display antitumor activity through diverse mechanisms which can be generally organized into 10 categories in this section.  Table 1 below provides a number of chalcones and specific tumor cell lines against which they show direct cytotoxicity, and Table 2 summarizes the inner mechanisms of antitumorous effect of each chalcone.

### 3.1. Cytotoxicity

In most cases, cell cytotoxicity was monitored using the MTT or MTS assay. Treatment of cells with chalcones can cause a decrease in cell viability, and then the cells undergo rapid necrosis or activate a programmed death (apoptosis). However, almost none of the investigations further reported the process and the way of cell death induced by chalcones in Table 1.

In this table, chalcones exhibit cytotoxicity against a number of cell lines from a wide range of tumors, mainly including leukemia, hepatoma, breast cancer, colorectal cancer, stomach cancer, prostate cancer, and epidermoid carcinoma. Furthermore, the IC_50_ values of the majority of compounds were below 50 *μ*M, especially that of chalcone **7** reaching nanomolar concentration, indicating the significant cytotoxicity of chalcones.

In spite of the strong activity of chalcones, they do not always show the ideal selectivity between normal cells and tumorous cells. Also, the underlying mechanisms causing cell death remain to be elucidated in detail.

### 3.2. Apoptosis

Apoptosis is the process of programmed cell death that may occur in multicellular organisms. It involved two central pathways in general. One involves the stimulation of death receptors (DRs) of the tumor necrosis factor (TNF) receptor superfamily, and the other is the mitochondrial pathway initiated by Bcl-2 family proteins [36].

#### 3.2.1. Death Receptor-Mediated Pathway

Death receptors are the members of TNF receptor superfamily, and all contain an intracellular death domain that serves to recruit adapter proteins such as TRADD and FADD and the initiator caspase-8. Caspases are cysteine proteases that cleave proteins at aspartic acid residues contained within a tetrapeptide recognition motif. There are two types of apoptotic caspases, namely, initiator caspases and effector caspases. Initiator caspases (caspase-2, -8, -9, -10) mediate the transduction of death signal and promote the formation of death-inducing signaling complex. Effector caspase (caspase-3, -6, -7) in turn cleave key cellular proteins or activate other degradation enzymes to execute the apoptosis [37]. Cleavage of the DNA repair-associated enzyme poly (ADP-ribose) polymerase (PARP) is accepted as a prominent marker of apoptosis [38].

Chowdhury et al. investigated 11 compounds for their cytotoxicity and apoptosis-inducing activity in four human tumor cell lines, HSC-2, HSC-3, HSG and HL-60. Among them, isoliquiritigenin exhibited higher cytotoxicity against tumor cells than normal cells with a tumor-specific index of 4.0. It induced internucleosomal DNA fragmentation and activation of caspase-3, -8 and -9 dose-dependently [4]. Xanthoangelol exhibited concentration-dependent cytotoxicity against human neuroblastoma IMR-32 and leukemia Jurkat cells through activation of caspase-3 [39]. Similarly, millepachine activated caspase-3 in HeLa-C3 cells within 36 h at the concentration of 5 *μ*M [25]. In addition, panduratin A, isolated from an extract of *Kaempferia pandurata*, was a strong inhibitor of cyclooxygenase-2 (COX-2) in RAW264.7 cells. This property was also revealed against HT-29 colon cancer cells because its treatment with panduratin A led to cleavage of PARP along with the decrease of procaspase-3 [40].

TNF-related apoptosis-inducing ligand (TRAIL), a member of TNF superfamily, selectively induces apoptosis in cancer cells with no toxicity to normal tissues [41]. However, more than 50% of tumor cells are resistant to TRAIL at different and/or multiple levels in the signaling pathway. Combined treatment with other therapeutic agents is a potential way to circumvent resistance and resensitize tumors. Compounds of natural origin play a specific role in this activity [42]. For example, butein exhibited significant synergism with TRAIL and sensitized TRAIL-resistant human leukemia U937 cells to apoptosis. It also increased caspase-3 activity and expression of DR5 [43]. In addition, butein, isobavachalcone, xanthohumol, licochalcone A and 2′,6′-dihydroxy-4′-methoxychalcone can markedly enhance TRAIL-mediated apoptosis and cytotoxicity in prostate cancer LNCaP cells. The combination of butein and TRAIL increased the percentage of cell death by 36.82–81.97% compared to TRAIL or butein alone. And the percentage of that was 24.47%–85.90% for isobavachalcone, 26.22%–93.20% for xanthohumol, 25.36–89.32% for licochalcone A, and 24.65%–67.59% for 2′,6′-dihydroxy-4′-methoxychalcone [44, 45]. Also, the augment of TRAIL-induced apoptosis by isobavachalcone, xanthohumol and licochalcone A was verified in HeLa cells by increased expression of DR5 [46].

Activity-guided fractionations of the extract of *Catimbium speciosum* afforded six compounds with DR5 inducing activity. The combination of cardamonin, the most active compound, and TRAIL synergistically enhanced TRAIL-induced apoptosis of TRAIL-resistant human colon cancer (DLD1/TR) and human gastric adenocarcinoma cells. The mechanism of the apoptosis induction in DLD1/TR cells was associated with the upregulation of DR4 and DR5 due to activation of caspase-8, 9, and 3. Moreover, upregulation of DR5 by cardamonin was accompanied with the induction of CCAAT/enhancer-binding protein-homologous protein (CHOP) [47]. Similarly, the combination of suboptimal concentration of isoliquiritigenin and TRAIL drastically enhanced apoptosis in colon cancer HT-29 cells compared with the single treatment of isoliquiritigenin. Isoliquiritigenin upregulated the level of DR5 and the combination of two agents induced apoptosis synergistically through activation of caspase-8, -10, -9, and -3 [48].

#### 3.2.2. Mitochondrial-Mediated Pathway

Mitochondrial pathway is regulated by Bcl-2 family of proteins. These proteins can dominate the permeability of mitochondrial outer membrane, and they are either proapoptotic (Bax, Bak, and Bad) or antiapoptotic (Bcl-2, Bcl-xL, and Bcl-W). The changes of mitochondrial membrane induced by proapoptotic factors result in cytochrome c release and the production of reactive oxygen species (ROS), while antiapoptotic members reside in the outer mitochondrial membrane and counter these effects. Death receptor and mitochondrial-mediated pathways are intimately connected. Cytochrome c released into cytoplasm is associated with Apaf-1 and then activates procaspase-9 to assemble the apoptosome [36, 49]. Thus, downstream caspase cascade is activated.

The Kava extract and flavokawains A, B, and C showed strong antiproliferative and apoptotic effects on human bladder cancer cells characterized as low grade or high grade. Flavokawain A induced a loss of mitochondrial membrane potential and release of cytochrome c in an invasive bladder cancer cell line T24, which were associated with an increase in Bax/Bcl-xL ratio and Bax confirmation change in T24 cells. Furthermore, it was observed that Bax protein was, at least in part, required for the apoptotic effect of flavokawain A on primary mouse embryo fibroblasts, and Bax inhibitory peptide P5 attenuated the antiproliferative effect of flavokawain A on T24 cells. In addition, flavokawain A downregulated the expression of X-linked inhibitor of apoptosis and survivin and remarkably inhibited growth of bladder tumor cells in a nude mice model and in soft agar [6]. 

Also, flavokawain B exerted the apoptotic action on HCT116 cells through ROS generation and GADD 153 upregulation, which led to mitochondria-dependent apoptosis. The upregulation of GADD 153 was linked with mitochondrial dysfunction and altered expression of Bcl-2 family members. Besides, flavokawain B was able to induce G2/M accumulation as well as autophagy [50].

Tang et al. compared the inhibitory activity of flavokawain B on the growth of androgen receptor- (AR-) negative, hormone-refractory prostate cancer cell lines DU145 and PC-3 with the AR-positive, hormone-sensitive cell lines LAPC4 and LNCap. This compound was 4- to 12-folds as effective on the former cell lines as the latter ones, with minimal effects on normal prostatic epithelial and stromal cells. It induced apoptosis through upregulation DR5, Bim and Puma expression and downregulation of XIAP, and surviving expression, which resulted in activation of the Bax-initiated mitochondria pathway. It was observed that significant increase of Bim expression played a critical role in flavokawain B-mediated apoptosis and flavokawain B exerted a synergistic apoptotic effect when combined with TRAIL. Moreover, flavokawain B was demonstrated to inhibit tumor growth and increase Bim expression in the mice bearing DU145 xenograft tumors [51]. Flavokawain B was also effective in human squamous carcinoma *in vitro* and* in vivo*. Both death receptor and mitochondrial pathways were involved, which was evidenced by the activation of caspase-9, -3, and -8 and cleavage of PARP and Bid, as well as changes of Bcl-2 and Bax levels. It induced G2/M phase block via reduction of cyclin A, cyclin B1, Cdc2, and Cdc25C and augmentation of p21/WAF1, Wee1, and p53. Moreover, tumor metastasis-related proteins, such as matrix metalloproteinase-9 (MMP-9) and urokinase plasminogen activator (u-PA), were decreased and the endogenous inhibitors tissue inhibitor of MMP-1 and plasminogen activator inhibitor-1 (PAI-1) were increased. In addition, the *in vivo* effect on tumor was shown in KB cell-derived tumor xenografts in nude mice and the induction of DNA fragmentation was observed [52].

Recently, Meng et al. reported that *Ashitaba* chalcone, a prenylated chalcone isolated from *Ashitaba*, could induce expressions of caspase-3 and Bax protein and inhibit proliferation of mouse hepatocarcinoma cells [53].

2′,4′-dihydroxy-3′,5′-dimethyl-6′-methoxychalcone exhibited cytotoxicity against human chronic leukemia K562 cells *in vitro* with an IC_50_ value of 14.2 *μ*M and was found to be able to induce apoptosis of K562. Further analysis on the mechanism revealed that this compound was able to downregulate the level of Bcl-2 protein without any effects on the expression of Bax protein [54].

Isoliquiritigenin led to dose-dependent decrease of the viable cell numbers and induction of apoptosis in DU145 and MAT-LyLu rat prostate cancer cells. It upregulated the levels of membrane-bound FasL, Fas, cleaved caspase-8, truncated Bid, Bax, and Bad in DU145 cells. Also, it increased the percentage of cells with depolarized mitochondrial membranes in a concentration-dependent manner. Additionally, isoliquiritigenin induced the release of cytochrome c and Smac/Diablo from the mitochondria into the cytoplasm and upregulated the levels of cleaved caspase-9, -7, and -3 and PARP [55].

Subsequently, Motani et al. conducted another study to clarify the mechanism of xanthoangelol apoptotic action in neuroblastoma cells. It increased ROS and induced the release of cytochrome c and activation of caspase-9 in IMR-32 cells. DJ-1 protein was involved in the apoptosis and was downregulated by xanthoangelol, which resulted in the loss of antioxidant function and promotion of apoptosis. Moreover, xanthoangelol exhibited cytotoxic effect on both drug-resistant LA-N-1 and NB-39 cells and drug-sensitive IMR-32 and SK-N-SH cells [56].

Boesenbergin A induced apoptosis via the regulation of the expression of Bcl-2 and Bax and significantly increased caspase-3, -7, -9, and -8. Moreover, it induced MMP disruption and increased ROS formation. In addition, the HSP 70 protein, which is well known to prevent protein aggregation caused by the oxidative stress in tumor cells, decreased concomitantly [57].

Isolespeol exhibited the strongest activity in human liposarcoma cells SW 872 with an IC_50_ value of 3.8 *μ*M. It caused the loss of mitochondrial membrane potential and increased expression of Fas, FasL, and p53 and led to the regulation of Bcl-2 family members and subsequent activation of caspase-3 and -9 followed by cleavage of PARP [26].

Also, isobavachalcone and xanthoangelol H demonstrated the cytotoxicity against neuroblastoma cell lines IMR-32 and NB-39 with no effects on normal cells. Isobavachalcone induced apoptosis which was evidenced by the significantly reduction of procaspase-3 and -9 and subsequently increase of cleaved caspase-3 and -9 levels in both neuroblastoma cell lines. Moreover, Bax was induced markedly by isobavachalcone as well [58].

In addition, xanthohumol significantly reduced proliferation of HCT116-derived colon cancer cell line 40–16. Further experiments clarified that xanthohumol induced apoptosis through downregulation of Bcl-2 and activation of the death receptor pathway, indicated by activation of caspase-3, -7, -8, and -9 [59]. 

### 3.3. Apoptosis under Hypoxia

Tumor hypoxia is the situation where tumor cells have been deprived of oxygen. As a tumor grows, it rapidly outgrows its blood supply, leaving portions of the tumor with regions where the oxygen concentration is significantly lower than in healthy tissues. In case of solid tumors, most tumor cells exist in chronic hypoxia, little nutrients, and low pH condition. Many genes, related to metabolism of energy, apoptosis and angiogenesis, are regulated in these hypoxic cells [60]. Hypoxia inducible factor (HIF) is recognized as a key protein in hypoxic signaling transduction. HIF-1 is thought to play important roles in tumor proliferation, angiogenesis, and metastasis [61, 62].

Six Diels-Alder adducts including sanggenons O and C from Mori Cortex Radicis, kuwanons J, Q, R, and V from *Morus bombycis*, as well as isoliquiritigenin, were found to strongly inhibit HIF-1 [63, 64]. Sanggenon O and kuwanon J also exhibited dose-dependent inhibition of hypoxia-induced HIF-1*α* accumulation and inhibited vascular endothelial growth factor (VEGF) secretion in Hep3B cells [63]. Xanthohumol could reverse the hypoxia-induced upregulation of the triglyceride synthesis and cancel the appearance of lipid droplets in the cytoplasm completely. It suppressed the proliferation of HT-1080 human fibrosarcoma in hypoxic condition as well as the motility enhanced by hypoxia, which demonstrated the potent and specific activities of xanthohumol against cancerous cells in hypoxic condition [65].

### 3.4. NF-*κ*B Pathway

NF-*κ*B, short for nuclear factor kappa-light-chain enhancer of activated B cells, is another transcription factor which is emerging as a potential target. While in an inactivated form, NF-*κ*B resides in the cytoplasm complexed with the inhibitory protein I*κ*Bs. I*κ*B kinases (IKK) phosphorylate I*κ*B proteins, which results in proteasome-assisted degradation of I*κ*B and then the translocation of NF-*κ*B into nucleus. The activated NF-*κ*B binds itself to specific sequence of gene, leading to a change of cell function [66, 67]. In many kinds of tumor cells, NF-*κ*B is constitutively active, and it keeps the cell proliferating and protects cells from the proapoptotic action of antitumor agents [68]. Accordingly, blocking NF-*κ*B is considered as an important strategy in cancer therapy.

Butein inhibited TNF-induced NF-*κ*B activation in a dose- and time-dependent manner, suppressed the NF-*κ*B activation induced by various carcinogens and inflammatory agents, and inhibited the NF-*κ*B reporter activity induced by TNFR1, TRADD, TRAF2, NIK, TAK1/TAB1, and IKK-*β*. It directly inhibited IKK activation, which subsequently blocked I*κ*B*α* phosphorylation and degradation. The direct inactivation of IKK by butein involved cysteine residue 179, which was associated with the suppression of phosphorylation and the nuclear translocation of p65. Butein also suppressed the expression of NF-*κ*B regulated gene products involved in antiapoptosis (IAP2, Bcl-2, and Bcl-xL), proliferation (cyclin D1 and c-Myc) and invasion (COX-2 and MMP-9), which resulted in potentiation of apoptosis induced by TNF as well as chemotherapeutic agents and suppression of cellular invasion [69].

Xanthohumol could enhance TNF-induced apoptosis in leukemia and myeloma cells, which was linked with downregulation of NF-*κ*B survival. Xanthohumol abrogated both constitutive and inducible NF-*κ*B activation, inhibited phosphorylation and degradation of I*κ*B*α*, and suppressed p65 nuclear translocation and NF-*κ*B-dependent reporter gene transcription. Inhibition of TNF-induced IKK activation and binding of p65 to DNA by xanthohumol could be reversed by a reducing agent, which indicated the role of cysteine residue. Thus, xanthohumol potentiated apoptosis in leukemia cell through modification of cysteine residues in IKK and p65 and thereby inhibited NF-*κ*B activation pathway [70]. Also, xanthohumol induced significant apoptosis of two hepatocellular carcinoma cell lines (HepG2 and Huh7) at 25 *μ*M and showed antiproliferation and antimigration activities, inhibited TNF-induced NF-*κ*B activity, and interleukin-8 at even lower concentrations. Nevertheless, the viability of primary human hepatocytes was not affected by the concentration of xanthohumol as high as 100 *μ*M [71].

Moreover, xanthohumol exhibited strong growth-inhibitory and apoptosis-inducing activity in both hormone-sensitive and refractory human prostate cancer cell lines. It induced apoptosis through enhancement of binding of V-FITC to cancer cells, cleavage of PARP-1, activation of procaspase-3, -8, and -9, mitochondrial depolarization, and release of cytochrome c. The induction of apoptosis was associated with inhibition of Akt (protein kinase B), NF-*κ*B and mTOR prosurvival signaling proteins, and NK-*κ*B mediated Bcl-2 pathway [72]. Xanthohumol also exhibited its potential through activation of Bax, p53, and downregulation of NF-*κ*B activation in human benign prostate hyperplasia epithelial BPH-1 cells [73]. In addition, panduratin A was found to be an inhibitor of NF-*κ*B translocation activated by TNF, and it caused growth inhibition in A549 cells and G2/M phase block [74].

### 3.5. Chemoprevention

Estrogen exposure is considered to be closely linked to the mutation and proliferation of breast cancer. Aromatase is the enzyme that synthesizes estrogen and its inhibitors can either block the production of estrogen or the action of estrogen on receptors [75, 76]. Butein can modulate estrogen metabolism and inhibit proliferation of breast cancer cells as an inhibitor of aromatase with an IC_50_ value of 3.75 *μ*M. Further enzyme kinetic study revealed that butein acted on aromatase with a mixed type of inhibition and the *K*_*i*_ value was 0.32 *μ*M. The cell number increased by 10 nM -testosterone treatment was remarkably reduced by 5 *μ*M butein, and the administration of flutamide could not reverse the effect [77].

Isoliquiritigenin exhibited either antitumorigenic activity or estrogen receptor *α*-(ER*α*-) dependent growth promoting effects on breast cancer cells. Maggiolini et al. studied this paradox in detail using the hormone-sensitive MCF-7 breast cancer cells and the steroid-independent HeLa cells as model systems. It could transactivate the endogenous ER*α* due to the downregulation of ER*α* protein levels and upregulation of pS2 mRNA. This compound was also proved to be an estrogenic agonist of ER*α* and ER*β*. As a biological counterpart, low and intermediate concentrations of isoliquiritigenin stimulated the proliferation of MCF-7 cells, whereas its high levels became cytotoxic even in steroid-receptor negative HeLa cells [78].

Azoxymethane- (AOM-) induced aberrant crypt focus (ACF) formation model in mice is often used to evaluate the potency of chemopreventive agents against colon cancer. Administration of isoliquiritigenin in drinking water significantly suppressed AOM-induced ACF formation, with an inhibitory ratio of 37.3%. Isoliquiritigenin administrated along with diet also significantly inhibited AOM-induced colon carcinogenesis [79]. Chin et al. demonstrated that isoliquiritigenin prevented colon and lung tumors induced by 1,2-dimethylhydrazine in mice when administered at a dose of 300 mg/kg [80]. It significantly decreased PGE_2_ production through downregulation of COX-2 expression and suppressed NO production by decreasing iNOS protein expression in RAW264.7 mouse macrophage cells. Isoliquiritigenin-induced growth inhibition and apoptosis in mouse and human colon carcinoma cells might be due to the inhibition of both PGE_2_ and NO production. Moreover, it was reported that *in vivo* administration of isoliquiritigenin inhibited preneoplastic ACF induction in the male F344 rat colon [81]. Also, isoliquiritigenin was proved to induce phase 2 enzymes, including quinone reductase (QR) and gluthatione S-transferase in the rat liver tissue [82]. And it exhibited significant peroxynitrite scavenging activity. Similarly, 1,2-dihydroparatocarpin A was prevented from oxidative damage by inhibition of peroxynitrite and NO production [83, 84].

A series of compounds were isolated and characterized from an ethyl acetate-soluble fraction of stem exudates of *Angelica keiskei*. Researchers further investigated the inhibitory effects of these compounds on the induction of EBV-EA (Epstein-Barr virus early antigen) by TPA in Raji cells. Among these compounds, xanthoangelol F, xanthoangelol H, xanthoangelol, isobavachalcone, and 4-hydroxyderricin demonstrated strong inhibitory effects on EBV-EA induction. In-depth studies showed that xanthoangelol F and isobavachalcone were able to inhibit activation of (±)-(E)-methyl-2-[(E)-hydroxyimino]-5-nitro-6-methoxy-3- hexemide (NOR 1), an NO donor, as a primary screening test for antitumor-initiators [85]. Afterwards, Akihisa et al. isolated two new chalcones, xanthoangelols I and J, and both of them showed inhibitory abilities on EBV-EA induction by TPA and activation of NOR 1. Isobavachalcone was observed to inhibit growth of skin tumor cells in a two-stage carcinogenesis test in mouse skin using DMBA and TPA as the inhibitor and promoter, respectively [86]. Moreover, isobavachalcone significantly inhibited the production of NO in lipopolysaccharide-activated macrophages [87]. And it is effective in preventing lipid peroxidation in rat liver microsomes and mitochondria induced by NADPH, ascorbate, CCl_4_, and so forth, [88, 89].

Furthermore, xanthohumol could modulate carcinogen metabolism through inhibition of phase 1 Cyp1A (IC_50_ = 0.02 *μ*M) and induction of phase 2 NAD(P)H:quinone reductase (CD = 1.7 *μ*M). It was also tested for the potential to inhibit iNOS induction and COX-1 and COX-2 activities with IC_50_ values of 12.9, 16.6, and 41.5 *μ*M, respectively. Based on these results above, xanthohumol was additionally tested in an organ culture model using mouse mammary glands. It significantly inhibited chemically induced preneoplastic lesions with an IC_50_ value of 0.02 *μ*M [90].

ROS can cause DNA damages, which subsequently results in cancer initiation. In order to search for chemopreventive agents of natural origin to destroy ROS, Kaur et al. studied the fraction “Rlicca” isolated from *Glycyrrhiza glabra*, which was further identified as isoliquiritin apioside, for its effect against genotoxicity induced by H_2_O_2_ and 4NQO in *Escherichia coli* PQ37 using SOS chromotest and in human peripheral blood lymphocytes using the Comet assay. In SOS chromotest, “Rlicca” (191 *μ*M) was found to be effective in decreasing the SOS inducing potency of H_2_O_2_ (1.0 mM) and NQO (20 *μ*g/mL) by 83.72% and 68.77%, respectively. In the Comet assay, “Rlicca” of all the doses reduced the tail moment induced by H_2_O_2_ (25 *μ*M) and NQO (5 *μ*g/mL) by 88.04% and 76.64%, respectively [91]. 

The formation of Bcr-Abl oncogene is due to fusion between Abl tyrosine kinase gene and Bcr gene and a constitutively active Bcr-Abl tyrosine kinase plays a crucial role in the proliferation of chronic myelogenous leukemia cells. Xanthohumol showed *in vitro* activity against Bcr-Abl(+) cells and clinical samples and retained cytotoxicity in imatinib mesylate-resistant K562 cells. It induced apoptosis, increase of p21 and p53 expression, and decrease of surviving levels in K562 cells. It also markedly inhibited Bcr-Abl expression at both mRNA and protein levels and caused elevation of intracellular ROS. Furthermore, xanthohumol inhibited leukemia cell invasion, metalloprotease production, and adhesion to endothelial cells, potentially preventing* in vivo *life-threatening complication of leukostasis and tissue infiltration by leukemic cells. Distinct from that of imatinib, this mechanism targets Bcr-Abl mRNA and protein and thus does not appear to be circumvented by Abl kinase mutations [92].

### 3.6. Cell Signal Transduction

Protein kinases or phosphokinase are enzymes that transfer *γ*-phosphate from ATP to an amino acid residue in proteins. Protein phosphorylation plays a prominent role in a wide range of cellular signal transduction pathways. And aberrant expression or activation of kinases can result in perturbation of relevant cellular processes, including cell growth, proliferation, and death. Protein kinases family is divided into tyrosine kinases and serine/threonine kinases.

Isoliquiritigenin was found to inhibit proliferation of prostate cancer cells through inhibition of ErbB3 signaling which belongs to tyrosine kinases. It reduced the protein and mRNA levels of ErbB3 dose dependently and showed no effect on HRG protein levels. It also inhibited HRG-*β*-induced tyrosine phosphorylation of ErbB3, recruitment of the p85 regulatory subunit of PI3K to ErbB3, and Akt phosphorylation in DU145 cells [93].

Recently, Jing et al. found isobavachalcone could abrogate Akt signaling significantly, which played a critical role in its antiproliferative effects on human cancer cells. Results showed that this chalcone could bind to the ATP-binding pocket of Akt. Furthermore, isobavachalcone markedly abated Akt phosphorylation at Ser-473 and Akt kinase activity and thereby inhibited Akt downstream substrates and induced apoptosis [94].

It was reported that butein exhibited antiproliferative effects against tumor cells through suppression of the signal transducer and activator of transcription 3 (STAT3) activation pathway. Butein inhibited both constitutive and interleukin-6-inducible STAT3 activation in multiple myeloma cells through inhibition of activation of the upstream kinases c-Src, Janus-like kinase1 (JAK1), and JAK2. Also, the induction of tyrosine phosphatase SHP-1 was involved in the action of this chalcone. It resulted in apoptosis by downregulating expression of STAT3-regulated gene products such as Bcl-xL, Bcl-2, cyclin D1, and Mcl-1. Butein markedly potentiate the apoptotic effects of thalidomide and velcade in multiple myeloma cells. Moreover, overexpression of constitutive active STAT3 significantly reduced the butein-induced apoptosis [95]. 

The ethanol extract of *Helichrysum maracandicum* exhibited strong antiproliferative activity against SENCAR mouse skin transformed cells and SENCAR mouse skin transformed tumor cells *in vitro*. Activity-guided fractionation of the extract led to the identification of isosalipurposide and its aglycone, naringenin chalcone as active substances. Further *in vivo* assay of two-stage carcinogenesis on mouse skin revealed that epidermal application isosalipurposide resulted in delayed formation of papillomas. It was also observed that administration of naringenin chalcone or isosalipurposide inhibited the expression of p38 mitogen-activated protein (MAP) kinase [96].

Transforming growth factor-*β* (TGF-*β*) superfamily is known to regulate cellular tissue regeneration, differentiation, immune surveillance, angiogenesis, and apoptosis. Isocordoin was investigated on its effect on TGF-*β*1 in mice with L5178Y lymphoma. Results showed that it exhibited antitumor activity through modulation of TGF-*β*1 concentration in plasma and ascitis of lymphoma-bearing mice [97].

Alkaline phosphatase (ALP) dephosphorylates proteins which are involved in cell growth and differentiation, apoptosis, and cell migration. Abnormal expression of ALP isoenzymes has been found in malignant tissues, being often established as a useful prognostic indicator. Guerreiro et al. reported that incubation with 10 *μ*M xanthohumol (IC_50_) in MCF-7 cells decreased proliferation and RNA and protein expression of intestinal ALP, but not TNS-ALP (tissue nonspecific ALP). The modulation of ALP by xanthohumol may be relevant to the development of therapeutic and preventive strategies against breast cancer [98].

### 3.7. Antiinvasion and Antimetastasis

Tumor invasion and metastasis are considered as the main therapeutic challenges in oncology. Invasion is the result of a disequilibrium between the expression of invasion promoter and invasion suppressor genes, in a sense that carcinomas either upregulate the promoters or downregulate the suppressors [99]. The tumor-secreted proteases not only degrade barriers (basement membrane and interstitial matrix) against invasion, but also cleave off cell surface glycoproteins which are essential for cell-cell/matrix adhesion. Malignant tumor cells intravasate into the blood vessels by passive transport and intravasation into new environment, which establishes a metastasis [100]. How to overcome chemotherapy resistance of invasion and metastasis is a big challenge in oncology.

Xanthohumol was able to inhibit the invasion of MCF-7/6 and of T47-D breast cancer cells. Aggregation assay showed that xanthohumol could stimulate aggregation of MCF 7/6 cells in suspension via restoring the function of E-cadherin/catenin complex, a powerful invasion suppressor [101]. Moreover, in T47-D cells xanthohumol stimulated the transcription and translocation of the endoplasmic reticulum chaperone GRP78, a maker for endoplasmic reticulum stress. The stress transducers PERK, IRE1 and ATF6 were activated as well. Xanthohumol motivated the phosphorylation of eIF2, the splicing of XBP1 and the cleavage of ATF6. The sustained endoplasmic reticulum stress led to changes of apoptosis-related factors and the susceptibility of xanthohumol-induced apoptosis appeared to associate with the GRP78 expression levels. Accordingly, xanthohumol inhibited invasion of breast cancer cells through stimulation of the E-cadherin mediated cell-cell adhesion and apoptosis in the number of invasive cells by promoting a chronic stress condition [102].

Also, it was reported that xanthohumol targeted the endoplasmic reticulum and activated the unfolded protein response and thus induced apoptosis of chronic lymphocytic leukemia cells from 18 patient samples. The apoptosis was accompanied by induction of GRP78 and Hsp70 protein levels and by sustained phosphorylation of eIF2*α*, indicating the involvement of PERK. It also induced splicing of the XBP1 mRNA without activation of ATF6. In addition, xanthohumol showed proteasome inhibitory activity [103] and exerted similar effects on B-cell acute lymphocytic leukemia cells *in vitro* and in all xenografts mouse model. Treatment with xanthohumol impaired cell migration and invasion, with the contaminant downregulation of FAK, Akt, and NF-*κ*B expression [104].

Ngameni et al. studied the inhibitory effects of isolates from the twig of *Dorstenia turbinate* on MMP-2 secretion in brain tumor-derived U87 glioblastoma cells. Kanzonol C and 4-hydroxylonchocarpin exhibited strong inhibitory activities comparable to the documented MMP secretion inhibitors CHL and EGCg [105]. Ngameni et al. found that isobavachalcone, paratocarpin C, stipulin, and dorsmannin A displayed even higher inhibitory effects on MMP secretion than 4-hydroxylonchocarpin and kanzonol C as well as CHL and EGCg, with maximum inhibition ranging from 60% to 80% at 250 *μ*M [106].

Bioassay-directed fractionation of the extract from the roots of *Angelica keiskei* yielded two active compounds, xanthoangelol and 4-hydroxyderricin. Studies on xanthoangelol showed that it inhibited tumor growth in LLC-bearing mice as well as lung metastasis and prolonged survival time in carcinectomized mice at a daily dose of 50 mg/kg. Treatment with 50 or 100 mg/kg daily also inhibited liver metastasis and the growth of metastasized tumor cells [107]. Similarly, 4-hydroxyderricin at a dose of 50 mg/kg × 2/day orally inhibited the tumor growth in subcutaneous LLC-implanted mice and inhibited the lung metastasis and prolonged the survival time in mice after the removal of subcutaneous tumors by surgical operation. It also controlled the reduction of lymphocytes, CD4^+^, CD8^+^, and natural killer-T (NK-T) cells in the spleen of tumor-removed mice [108]. The antitumor and antimetastatic activity of xanthoangelol and 4-hydroxyderricin may be associated with inhibition of DNA synthesis, inhibition of tumor-induced neovascularization by reducing formation of capillary-like tubes in human umbilical vein endothelial cells (HUVECs), and suppressing binding of VEGF to HUVECs [107, 108].

When examined in a pulmonary metastasis model of mouse renal cell carcinoma, isoliquiritigenin exhibited significant activity without any weight loss or leukocytopenia. It suppressed proliferation of carcinoma cells *in vitro*, potentiated NO production by lipopolysaccharide-stimulated macrophages, and enhanced cytotoxicity of splenic lymphocytes *in vitro*. In addition, it prevented severe leukocytopenia induced by administration of 5-fluorouracil [109].

### 3.8. Regulation of Cell Cycle

Cell cycle is a series of complicated and fine adjustment processes which lead to cellular differentiation, growth, and programmed death. And aberrant regulation of cell cycle is regarded as one of the most distinguishing marks. Cyclin-dependent kinases (CDKs) are a family of proteins that plays an essential role in regulating cell cycle, and activation of CDK is controlled by cyclins and cyclin-dependent kinase inhibitors (CKIs) [110]. 

Yun et al. conducted a study to confirm tumor cell growth inhibitory effect of panduratin A in androgen-independent human prostate cancer cells PC-3 and DU145. Panduratin A treatment inhibited cell growth with an IC_50_ of 13.5–14 *μ*M and had none to little effect on normal human prostate epithelial cells. It mediated apoptosis via both mitochondrial and death receptor pathways. Treatment by panduratin A led to G2/M phase arrest in a dose-dependent manner by modulating expression of G2/M regulatory molecules, including induction of p21/WAF1 and p27/Kip1 and downregulation of CDKs 2, 4, and 6 as well as decrease in cyclins D1 and E [111]. Similarly, isoliquiritigenin exhibited significant inhibitory effects against prostate cancer cell lines DU145 and LNCaP and caused S and G2/M phase arrest due to the expression enhancement of GADD153 mRNA and protein associated with cell cycle arrest. This compound also stimulated transcriptional activity of GADD153 promoter dose dependently [112].

Isoliquiritigenin was demonstrated to inhibit the proliferation of U87 glioma cells *in vitro*, and it caused cell cycle arrest at S and G2/M phases and induced caspase-mediated apoptosis pathway. Further examination showed p21/WAF1 and p27 were upregulated [113]. Similar activity of isoliquiritigenin was also found in human lung cancer cells A549, in which cell cycle arrest at G2/M phase was observed. cDNA arrays and real-time quantitative RT-PCR revealed isoliquiritigenin enhanced p21/WAF1 expression with no change of p53 [114]. However, Hsu et al. observed that isoliquiritigenin inhibited proliferation through p53 and Fas/FasL apoptotic system in A549 cells. An ELISA assay showed that the significant increase of the expression of p53 and p21/WAF1 protein led to cell cycle arrest in the G1 phase [115].

Isoliquiritigenin caused a G2/M-phase arrest in HepG2 cells and upregulated p53, p21/WAF1, Fas/APO-1 receptor, Fas ligand, Bax, and NOXA. The enhancement of p21/WAF1, Fas/APO-1, Bax, and NOXA was decreased in HepG2 cells that lack functional p53. And when the transcriptional activity of p53 was blocked, HepG2 cells became significantly more resistant to isoliquiritigenin [116].

Rao et al. synthesized 10 structurally related 2′-oxygenated chalcone derivatives including 2′-hydroxy-2,3,4′,6′-tetramethoxychalcone. The *in vitro* cytotoxicity against the tumor cells such as Jurkat, U937 cells, and normal cells PHA stimulated primary peripheral blood mononuclear cells (PBMCs) were investigated. This chalcone showed strong inhibitory activity toward both Jurkat and U937 cell lines, with IC_50_ values of 1.7 *μ*M and 1.5 *μ*M, respectively. The growth of normal cells PHA stimulated PBMCs was also inhibited, but relatively less sensitive to that of tumor cells. Cell cycle analysis of U937 showed that this chalcone caused a G2/M arrest with a concomitant decrease of cells in the S phase of cell cycle [117]. This chalcone was also a selective cytotoxic agent in human lung cancer cells. Among the 3 lungs cancer cell lines examined, 2′-hydroxy-2,3,4′,6′-tetramethoxychalcone was more sensitive to A549 cells than H1299 and H1355. In A549 cells, it induced G1 cell cycle arrest and upregulated tumor suppresser genes p21 and p53 and downregulated cell-cycle regulatory proteins phosphorylation of cdc2 (Tyr^15^ and Tyr^161^) and Rb (Ser^795^ and Ser^807/811^), which may be one of its molecular mechanisms. In addition, *in vivo* data demonstrated that this chalcone acted as a tumor growth suppressing agent [118]. 

In a report published in 2004, licochalcone A was found to induce growth control and apoptosis on androgen-independent p53-null PC-3 prostate cancer cells. It induced modest level of apoptosis but caused strong influence on cell cycle progression arresting cells in G2/M through suppression of cyclin B1 and cdc2. They also found that licochalcone A inhibited phosphorylation of Rb, specially phosphorylation at S780 without effect on that of T821. Moreover, licochalcone A could downregulate the expression of transcription factor E2F, reduce cyclin D1, and downregulate CDKs 4 and 6 while increasing expression of cyclin E [119]. In addition, isocordoin was found to be able to block the cell cycle of cancer prostate cell PC-3, and the percentages of cells in G1, G2, and S phase were 26.0, 53.3, and 20.5, respectively [27].

### 3.9. Antiangiogenesis

Angiogenesis is a process producing new blood vessels by making use of preexist vessels. The growth and metastasis of most malignant solid tumors depend on angiogenesis, by which nutrients and oxygen needed for growth can be provided.

Zhu et al. studied the potential antiangiogenesis effects of 2′,4′-dihydroxy-3′,5′-dimethyl-6′-methoxychalcone *in vitro* as well as *in vivo*. It reversibly inhibited vascular endothelial growth factor receptor (VEGF receptor, KDR) tyrosine kinase phosphorylation with no influence on epidermal growth factor receptor tyrosine kinase phosphorylation. Mitogen-activated protein kinase and AKT activation of KDR signal transduction was inhibited as well. Systemic administration of this chalcone at nontoxic doses to nude mice resulted in inhibition of subcutaneous tumor growth of human hepatocarcinoma Bel7402 as well as lung cancer GLC-82 xenografts and a decrease of tumor vessel density [120].

The antiangiogenic property of xanthohumol was tested in an *in vitro* system. Fragments of placental vessels were cultured in the presence or absence of the compounds for 3 weeks in fibrin gels; xanthohumol induced a dose-dependent reduction of newly formed capillary growth at a concentration range of 0.5–10 *μ*M (IC_50_ value of 2.2 *μ*M). Xanthohumol also inhibited migration of HMEC-1 cells (immortalised human microvascular endothelial cell line) after wounding in a wound closure assay, with an IC_50_ value of 0.03 *μ*M. Moreover, it effectively inhibited microcapillary tube formation of HMEC-1 cells at 1, 5, and 10 *μ*M, respectively. Intravital microscopy was used as an *in vivo* model to investigate the effect of xanthohumol on tumor angiogenesis *in vivo*. Subcutaneous application of xanthohumol for 7 and 14 days, respectively, resulted in inhibition of the growth of breast tumor xenografts by 46% and 83% and reduction of the size of tumors by 30% and 56%, respectively, in comparison with the untreated control group. Concomitantly, xanthohumol treatment for 14 days reduced tumor-induced neovascularization by 33% in comparison with the untreated control group [121, 122].

Furthermore, xanthohumol showed antiangiogenic property in the growth of a vascular tumor *in vivo*. This compound repressed both the NF-*κ*B and Akt pathways in endothelial cells. It was also shown that xanthohumol interfered with several steps of the angiogenic process, including invasion and migration, growth, and formation of a network of tubular-like structures [121, 123]. Xanthohumol inhibited cell proliferation in acute and chronic myelogenous leukemia cell lines, which was linked with induction of apoptosis, reduction of VEGF secretion, decrease of invasion, and metalloprotease production. Accordingly, xanthohumol could prevent *in vivo* life-threatening complications of leukostasis and tissue infiltration [124]. In addition, xanthohumol at 10 *μ*M markedly decreased tumor cells viability and invasion activity and potentiated apoptosis of HUVEC (human umbilical vein endothelial cell) and SMC (human fetal aortic smooth muscles cell) cells. Xanthohumol of 5 and 10 *μ*M also led to a significant reduction of capillary-like structures, when HUVECs were cultured on growth factor reduced-Matrigel-coated plates. However, xanthohumol caused an increase of the number of cord structures when HUVECs were cocultured with SMC. Furthermore, incubation with xanthohumol led to the reduction of activity of NF-*κ*B in both cell types [125].

### 3.10. Other Targets

#### 3.10.1. Fibroblasts

Samoszuk et al. investigated whether butein could inhibit the clonogenic growth of small numbers of breast cancer cells co-cultured with fibroblasts *in vitro*, and fibroblasts are commonly believed to play an essential role in promoting the growth of breast cancer cells,. The clonogenic growth of primary breast cancer cells was significantly inhibited when co-cultured with fibroblasts pretreated with a fixed dose of butein [128].

#### 3.10.2. Redifferentiation

Isoliquiritigenin inhibited HL-60 cells proliferation and decreased the iROS (intracellular ROS) levels dose dependently without an increase of the lethality rate. The treatment with 10 *μ*g/mL isoliquiritigenin in 72 h led to a typical differentiated morphology in HL-60 cells, including a decrease of karyoplasmic ratio and an increase of kidney-shaped nuclear cells. In addition, the positive rate of CD11b and CD14 cells increased significantly and the NBT reductive activity increased 2- and 3-folds as compared to that of the control group. These results indicated isoliquiritigenin could induce monocytic differentiation in leukemia cells [126]. 

#### 3.10.3. DNA Topoisomerase II

Due to the rapidly proliferative property of tumors, the content of topoisomerase II they need is far above that of normal cells. Therefore, inhibition of topoisomerase II results in limitation of proliferation, which has become an efficient target [130]. Pauferrol A, a chalcone trimer with a cylcobutane ring, isolated from the stems of *Caesalpinia ferrea* Mart., exhibited strong inhibitory activity against human topoisomerase II with an IC_50_ value of 2.1 *μ*M and apoptosis activity in human leukemia HL-60 cells with an IC_50_ value of 5.2 *μ*M [129].

#### 3.10.4. Supplement for Cisplatin Chemotherapy

Isoliquiritigenin has the potential to act as a beneficial supplement during cisplatin chemotherapy. The single administration of isoliquiritigenin markedly reduced the size of the solid tumors in CT-26 cell-inoculated BALB/c mice. The treatment also caused the reduction of the viability and DNA synthesis of CT-26 murine colon cancer cells dose dependently. Isoliquiritigenin not only had no effects on the therapeutic efficacy of cisplatin but suppressed kidney damage caused by cisplatin, characterized by increases in serum creatinine and blood urea nitrogen, as well as liver damage characterized by increases in serum alanine aminotransferase and aspartate aminotransferase. Furthermore, repeated oral administration of isoliquiritigenin prior to cisplatin treatment significantly inhibited cisplatin-induced increases in serum nitric oxide and tissue lipid peroxidation levels and recovered depleted GSH levels in the tissues [127]. 

## 4. Conclusion

Tumors, both benign and malignant, constitute a group of well-known refractory diseases which have been baffling scientists for quite a long time. Up to date, medicinal scientists have made a lot of efforts trying to overcome this millennium problem, and as a result, many new chemical drugs have been developed. Although these drugs currently available are favorable in alleviation of the condition and adjuvant chemotherapy, most of them have a limited application because of their less well effectiveness, severe toxicity, and high manufacturing cost. Herbal medicines and natural products isolated therefrom, for example, flavonoids, are being highly expected due to their novel structure, distinctive mechanism, satisfactory effectiveness, and reliable safety. Many investigations have shown the advantageous activities and multifarious mechanisms of flavonoids. Chalcones (1,3-diaryl-2-propen-1-ones) are a kind of open chain flavonoids, which makes their structures more diversified than other types. In the past decades, a number of chalcones were proved to have antitumor potential either *in vitro* or *in vivo*, and a large proportion of underlying mechanisms have been explained. Based on the current studies, chalcones are highly multifunctional and their targets cover almost all of the actions of tumor cells, including growth, proliferation, invasion, and metastasis. Moreover, researchers have discovered several chalcones with significant antitumor activity both *in vitro* and *in vivo*, such as xanthohumol, isoliquiritigenin, and butein. These chalcones can be served as promising lead compounds for further drug development. However, it should also be noted that much of the pharmacological potential of chalcones is still not utilized due to some unclear mechanisms, and more working such as fundamental and clinical researches should be done to make best use of these natural products.

## Figures and Tables

**Table 1:** Chalcones and the corresponding cell lines inhibited by them.

Chalcones	Cell lines	Reference
Butein (**1**)	HSC-2, HSC-3, HSG, HL-60	[4]
Flavokawain B (**2**)	T24, KB, HCT116	[5]
Flavokawain C (**17**)	T24, PANC-1	[6, 7]
Pinostrobin chalcone (**3**)	KB, MCF7, Caski, MRC5	[5]
2′,4′-dihydroxy-3,4-dimethoxychalcone (**4**), 2′,4′-dihydroxy-2,3-dimethoxychalcone (**5**), 2′,4′-dihydroxy-4-methoxychalcone (**6**)	HL-60, SMMC-7721	[8]
2′,4′-dihydroxy-3′,5′-dimethyl-6′-methoxychalcone (**7**)	SMMC-7721, 8898, HeLa, SPC-A-1, 95-D, GBC-SD, MCF-7, SKBR-3, SW-480	[9, 10]
Stercurensin (**8**)	SW-480	[11]
Cardamonin (**9**)	SW-480, PANC-1	[11]
2′,4′-dihydroxychalcone (**10**)	26-L5, B16-BL6, LLC, A549, HeLa, HT-1080	[12]
4,4′-dihydroxy-2′-methoxychalcone (**11**)	26-L5, B16-BL6, LLC, A549, HeLa, HT-1080, SH-SY5Y	[12, 13]
Isoliquiritigenin (**12**)	26-L5, B16-BL6, LLC, A549, HeLa, HT-1080	[12, 14]
3′,4,4′-trihydroxy-2,6-dimethoxychalcone (**15**), 3′,6-dihydroxy-2,4,4′-trimethoxy-3-(3′′,4′′-dihydroxybenzyl)chalcone (**95**)	MCF-7/ADR, BEL-7402/5-FU	[15]
3′-formyl-2′,4′-dihydroxy-6′-methoxychalcone (**20**)	KB, MCF-7	[16]
Abyssinone A (**50**), B (**51**), C (**52**), D (**27**)	Caco2	[17]
5′′-(2′′′-hydroxyisopropyl-)-dihydrofurano-[2′′,3′′-B]-4,4′-dihydroxy-6′-methoxychalcone (**67**)	BGC-823	[18]
Flavokawin (**13**), 3′-geranylchalconaringenin (**29**), 5′-prenylxanthohumol (**30**), xanthohumol H (**31**), xanthogalenol (**33**), 4-methylxanthohumol (**34**), xanthohumol C (**53**), 1′′,2′′-dihydroxanthohumol C (**55**)	HeLa	[19]
Desmethylxanthohumol (**32**)	HeLa, DU145, PC-3	[20, 21]
Licochalcone A (**35**), C (**36**), E (**37**)	HT-1080	[14]
2′,4′,2,4-tetrahydroxy-3′-prenyl-6′-methoxychalcone (**38**)	HL-60, L1210, U937	[22]
Cyclokuraridin (**58**)	KB	[23]
3-hydroxy-4-methoxylonchocarpin (**60**)	A2780, B16, K562, LL/2, 7860, HeLa	[24]
4-methoxylonchocarpin (**61**)	HeLa, K562, LL/2, 7860, A2780, B16	[24]
Isobavachromene (**62**)	7860, A549, A2780, B16, HeLa, K562, LL2	[24]
Millepachine (**66**)	HepG2, C26, LL2, B16	[25]
5′-geranyl-2′,4′,4-trihydroxychalcone (**39**)	SW 872, HT-29, Hep3B, PLC5, Huh7, HepG2, COLO205	[26]
Isocordoin (**40**)	P388	[27]
Derricin (**41**), lonchocarpin (**59**)	CEM	[28]
Isobavachalcone (**42**)	HT-1080, MCF-7, MCF-7/ADR	[29]
4-hydroxyderricin (**47**)	KATO III	[30]
Xanthoangelol (**44**)	SW 872, HT-29, COLO205, Hep3B, PLC5, Huh7, HepG2, KATO III	[26, 30]
Isolespeol (**68**)	HT-29, COLO205, Hep3B, PLC5, Huh7	[26]
2′,4′-dihydroxy-3′-(1′′-geranyl)-6′-methoxychalcone (**28**), (−)-nicolaioidesin B (**71**), (±)-isopanduratin A1 (**72**), (1′R,2′S,6′R)-2-hydroxyisopanduratin A (**73**), panduratin A (**74**), (−)-isopanduratin A2 (**75**), (−)-hydroxypanduratin A (**76**), (±)-panduratin C (**77**), (±)-6-methoxypanduratin A (**78**),	PANC-1	[7]
Australisine A (**85**)	HCT-8, A2780	[31]
Australisine B (**79**), mulberrofurans E (**80**), F (**81**), mongolicin C (**83**), chalcomoracin (**84**)	A549, Bel7402, BGC-823, HCT-8, A2780	[31]
Mulberrofuran G (**82**)	A549, BGC-823, HCT-8, A2780	[31]
Australisine C (**86**)	A2780	[31]
Yunanensin A (**87**)	A549, Bel7402, BGC-823, HCT-8, A2780	[32]
Japonicone A (**94**)	HeLa, Bel7402, MCF-7/ADR, Bel7402/5-FU	[33]
3′,6-dihydroxy-2,4,4′-trimethoxy-3-(3′′,4′′-dihydroxybenzyl)chalcone (**95**)	MCF-7/ADR, BEL-7402/5-FU	[15]
2′,4′-dihydroxy-3′-(2-hydroxybenzyl)-6′-methoxychalcone (**96**)	KB, BC	[34]
Rhuschalcones I (**23**), II (**24**), III (**25**), V (**97**), VI (**98**)	HT-29, HCT-116	[35]
Rhuschalcone IV (**26**)	HT-29, HCT-116, SK-MEL-5, SK-MEL-28, UACC-62	[35]
Xanthohumol (**49**)	DU145, PC-3, HeLa, MCF-7/ADR, HT-1080	[19–21, 29]

**Table 2:** Chalcones and their mechanisms.

Chalcones	Mechanisms	Reference
Cardamonin (**9**)	Induction of apoptosis	[47]
Flavokawain A (**16**)		[6]
Flavokawain B (**2**)		[50–52]
*Ashitaba* chalcone (**43**)		[53]
Isolespeol (**68**)		[26]
Boesenbergin A (**65**)		[57]
Millepachine (**66**)		[25]
2′,6′-dihydroxy-4′-methoxychalcone (**14**)		[45]
2′,4′-dihydroxy-3′,5′-dimethyl-6′-methoxychalcone (**7**)	Induction of apoptosis	[54]
	Antiangiogenesis	[120]
Isoliquiritigenin (**12**)	Induction of apoptosis	[4, 48, 55]
	Induction of apoptosis under hypoxia	[64]
	Chemoprevention	[78–81]
	Cell signal transduction	[93]
	Antimetastasis	[109]
	Regulation of cell cycle	[112–116]
	Redifferentiation	[126]
	Supplement for cisplatin chemotherapy	[127]
Xanthoangelol (**44**)	Induction of apoptosis	[39, 56]
	Chemoprevention	[85]
	Antimetastasis	[107]
Isobavachalcone (**42**)	Induction of apoptosis	[44, 58]
	Chemoprevention	[85, 86]
	Cell signal transduction	[94]
	Antimetastasis	[106]
Xanthohumol (**49**)	Induction of apoptosis	[44, 59]
	Induction of apoptosis under hypoxia	[65]
	NF-*κ*B pathway	[70–73]
	Chemoprevention	[90, 92]
	Cell signal transduction	[98]
	Antiinvasion	[101–104]
	Antiangiogenesis	[121–125]
Panduratin A (**74**)	Induction of apoptosis	[40]
	NF-*κ*B pathway	[74]
	Regulation of cell cycle	[111]
Butein (**1**)	Induction of apoptosis	[43, 44]
	NF-*κ*B pathway	[69]
	Chemoprevention	[77]
	Cell signal transduction	[95]
	Fibroblasts	[128]
Licochalcone A (**35**)	Induction of apoptosis	[44]
	Regulation of cell cycle	[119]
Sanggenon O (**88**), C (**89**), kuwanon J (**90**), Q (**91**), R (**92**), V (**93**)	Induction of apoptosis under hypoxia	[63]
Naringenin chalcone (**19**), isosalipurposide (**18**)	Cell signal transduction	[96]
Xanthoangelol F (**48**)	Chemoprevention	[85]
Isoliquiritin apioside (**22**)		[91]
Xanthoangelol I (**64**), J (**49**),		[86]
Xanthoangelol H (**63**)	Induction of apoptosis	[58]
	Chemoprevention	[85]
1,2-dihydroparatocarpin A (**70**)	Chemoprevention	[83]
4-hydroxyderricin (**47**)	Chemoprevention	[85]
	Antimetastasis	[108]
2′-hydroxy-2,3,4′,6′-tetramethoxychalcone (**21**)	Regulation of cell cycle	[117, 118]
Isocordoin (**40**)	Cell signal transduction	[97]
	Regulation of cell cycle	[27]
Kanzonol C (**45**), 4-hydroxylonchocarpin (**56**)	Antimetastasis	[105]
Paratocarpin C (**53**), stipulin (**46**), dorsmannin A (**57**)		[106]
Pauferrol A (**99**)	DNA topoisomerase II	[129]
